# Characterization techniques for tobacco and its derivatives: a systematic review

**DOI:** 10.3389/fchem.2024.1402502

**Published:** 2024-07-05

**Authors:** Kai Shen, Liwei Xia, Kaixuan Jiao, Fanda Pan, Boka Xiang, Wei Zhou, Yuedian Shou, Xuefeng Gao, Shihao Hu, Haoyu Fang, Chen Xia, Xinru Jiang, Xiaoyuan Gao, Cuiyu Li, Ping Sun, Guangzheng Lu, Hu Fan, Tulai Sun

**Affiliations:** ^1^ Technology Center, China Tobacco Zhejiang Industrial Co. Ltd., Hangzhou, Zhejiang, China; ^2^ College of Chemical Engineering, Zhejiang University of Technology, Hangzhou, Zhejiang, China

**Keywords:** biomass, characterization techniques, derivatives, structure, tobacco

## Abstract

Biomass and its derivatives have broad applications in the fields of bio-catalysis, energy storage, environmental remediation. The structure and components of biomass, which are vital parameters affecting corresponding performances of derived products, need to be fully understood for further regulating the biomass and its derivatives. Herein, tobacco is taken as an example of biomass to introduce the typical characterization techniques in unraveling the structural information, chemical components, and properties of biomass and its derivatives. Firstly, the structural information, chemical components and application for biomass are summarized. Then the characterization techniques together with the resultant structural information and chemical components are introduced. Finally, to promote a wide and deep study in this field, the perspectives and challenges concerning structure and composition charaterization in biomass and its derivatives are put forward.

## 1 Introduction

In recent years, there has been a growing interest in the development and utilization of biomass and its derivatives for various applications across different fields (Tang et al., 2017; Karahan et al., 2020; Wang et al., 2021; Hou et al., 2022; Malode et al., 2023). These derivatives, derived from renewable biomass sources such as plant residues, agricultural waste, and algae, offer a sustainable and environmentally friendly alternative to conventional nanomaterials. The unique properties of biomass and its derivatives make them promising candidates for applications in catalysis (Chen et al., 2022; Zhang et al., 2023), energy storage (Tang et al., 2017; Sawant et al., 2022), environmental remediation (Chakraborty et al., 2022; Kumari et al., 2023). For example, biomass-derived nanoenzymes have shown great promise for catalyzing a wide range of chemical reactions with high efficiency and specificity (Ma et al., 2022; Sun et al., 2022; Yao et al., 2022; Yin et al., 2024; Yue et al., 2024). The unique nanostructure and surface chemistry of biomass-derived nanoenzymes allow for precise control over their catalytic activity and substrate specificity, making them ideal candidates for various applications. Researchers have successfully tailored the properties of biomass-derived nanoenzymes by modulating factors such as particle size, morphology, and surface functionalization to optimize their catalytic performance for specific reactions (Li et al., 2021; Ma et al., 2022; Yin et al., 2024).

Characterizing biomass and its derivatives is essential for understanding their structure-property relationships and optimizing their performance for specific applications. As these biomass and its derivatives exhibit complex morphologies, compositions, and surface chemistries, special characterization techniques are required to probe their structural features at the nanoscale level. In this systematic review, we focus on exploring the latest advancements in typical characterization techniques that enable in-depth analysis of biomass and its derivatives.

By employing the special characterization techniques, researchers can gain valuable insights into the size, shape, crystallinity, surface area, porosity, and functional groups of biomass and its derivatives (Zhou et al., 2022; Yuan et al., 2023; Zhong et al., 2023). Techniques such as X-ray diffraction (XRD), transmission electron microscopy (TEM) and scanning electron microscopy (SEM) play a crucial role in elucidating the structural and chemical component of biomass and its derivatives. Moreover, spectroscopic techniques such as energy-dispersive X-ray spectroscopy (EDS) and nuclear magnetic resonance (NMR) provide detailed information about the elemental composition and molecular structure. These techniques help researchers understand the mechanisms governing the synthesis, growth, and properties of biomass and its derivatives.

In this systematic review, tobacco was taken as an example to show the biomass and its derivatives. Also, typical characterization techniques are introduced in unraveling the structural, morphological, chemical, and properties of tobacco and its derivatives. By providing a comprehensive overview of these techniques and their applications in research of biomass and its derivatives, we seek to pave the way for future advancements in the development and optimization of sustainable nanostructures with tailored properties for diverse applications.

## 2 Biomass and the derived products

Tobacco is a typical biomass and has become one of the most important crops globally, extensively cultivated and consumed (Geist, 2021; Sifola et al., 2023). Its main usage lies in the production of various tobacco products, including cigarettes, cigars, pipe tobacco, and chewing tobacco (Banožić et al., 2020; Banožić et al., 2021; Huang et al., 2021; Manca et al., 2021). Nicotine, the main active ingredient in tobacco leaves, possesses stimulant effects but also carries potential health risks. Smoking is recognized as a leading cause of various serious health issues, including cancer, cardiovascular diseases, and respiratory disorders (Slomski, 2016; Banožić et al., 2020; Wu et al., 2020; Banožić et al., 2021; Manca et al., 2021; Yadav et al., 2024). Aiming at aiding smokers in overcoming nicotine addiction, tobacco is also utilized in the production of nicotine replacement therapy products, such as nicotine gum, patches, and inhalers (Charlton, 2004; Slomski, 2016).

**FIGURE 1:**
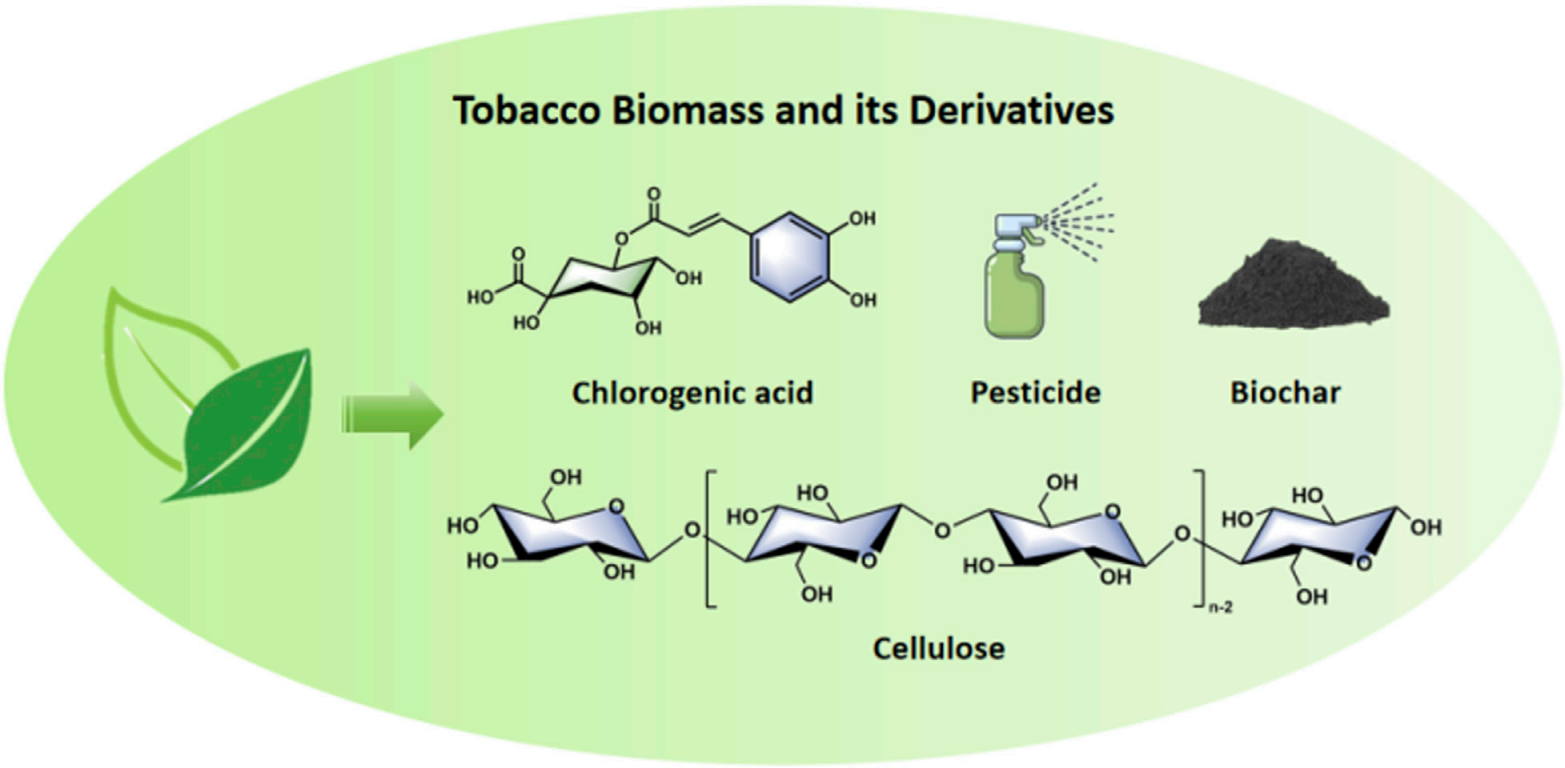
Carton model showing the applications of tobacco.

Apart from typical products, tobacco finds applications in the production of fertilizers, insecticides, and the extraction of beneficial bioactive compounds like phenols, solanesol, polysaccharides, and proteins for human health (Banožić et al., 2021; Bazok et al., 2021; Mandić et al., 2023). Additionally, as a biomass resource, tobacco can be utilized in the energy and chemical industries (Zhang et al., 2016), as shown in Figure 1, we list the application model diagram of tobacco. For example, Tobacco can be used to produce energy-related products such as biooil, biochar, and pyrolysis gas through pyrolysis (Lin et al., 2016; Yan et al., 2018; Ren et al., 2021; Muzyka et al., 2022). The chemical applications of tobacco biomass mainly involve the production of cellulose, hemicellulose, and lignin, which can be further hydrolyzed to yield a broad range of chemicals (Tuzzin et al., 2016; Sun et al., 2019). Yan et al. used a fluidized bed reactor to pyrolyze tobacco waste such as leaves and stems, successfully producing bio-oil containing aromatic compounds for use as liquid fuel. Sha et al. successfully prepared nitrogen-doped porous carbon with a specific surface area of 1,104 m^2^ g^−1^ by simple pretreatment of waste tobacco with melamine and applied it to electrochemical capacitors and carbon dioxide capture (Sha et al., 2015). The electrochemical performance study and carbon dioxide adsorption results proved that the method is feasible and has potential industrial applications. Using glutathione-assisted waste tobacco leaves as a precursor, Yu et al. achieved the synthesis of novel red fluorescence emission biomass-based carbon nanodots via a one-pot hydrothermal method. These carbon nanodots were then employed in the development of a sensing system capable of detecting and removing mercury ions, achieving an impressive removal rate of 99.4% (Yu et al., 2021; Yang et al., 2022b). Yang et al. successfully synthesized C-dots from waste tobacco stems by a simple pot hydrothermal method with the help of carbon black, and constructed a sensing system for the detection of tetracycline antibiotics, with a limit of detection for antibiotics of 1.328 nM (Yang et al., 2022a). As shown in Table 1, we have summarized the acquisition technologies and applications of tobacco biomass and its derivatives.

**TABLE 1:** Access to tobacco derivatives and their applications.

Techniques	Materials and chemicals	Applications	References
Pyrolysis	Biooil; porous carbon; pyrolysis gas	Liquid fuel; electrochemical capacitors	Lin et al. (2016), Yan et al. (2018), Ren et al. (2021), Muzyka et al. (2022)
Hydrothermal method	Biomass-based carbon nanodots	Sensing system	Yu et al. (2021), Yang et al. (2022a), Yang et al. (2022b)
Heating methods	Biomass-derived carbon materials; TS-biochar	Adsorption; achieving long-term stable restoration of heavy metal-polluted soils	Kazmierczak-Razna et al. (2017), Yu et al. (2021)
Liquid–liquid microextraction	Polyamines	Nucleic acid metabolism, protein synthesis, cell growth, and nicotine synthesis precursors	Cai et al. (2016), Deng et al. (2019)
Supercritical fluid extraction	Nicotine	Alkaloids and substances resistant to heat damage	Saginovich-Ikhsanov et al. (2019), Djapic (2022)
Solid Phase Extraction	Nitrosamines	The role of important pathogenic factors in Lung, Pancreatic, Oesophageal and Oral Cancers	Zhang et al. (2018), Ishizaki and Kataoka (2019)

## 3 The applications of tobacco biomass and its derived materials

Tobacco is widely cultivated as an important non-food cash crop worldwide, generating large quantities of biomass (Wang and Bennetzen, 2015; Jassbi et al., 2017), during the vegetative and post-harvest processes. When talking about the applications of tobacco, we are not limited to its use as a source of smoking. In fact, the composition of biomass is complex and diverse, and it has many derivatives, so tobacco plants have broader application prospects (Gui et al., 2024). Biologically, components such as nicotine in tobacco are not only a major component of smoking, but also possess medicinal uses. Nicotine is extracted for the preparation of smoking cessation products (Baker et al., 2016; Lindson et al., 2019), such as chewing gums and patches, which provide smokers with an effective means of quitting smoking. In addition, the bioactive compounds in tobacco can be used in the preparation of biopesticides to replace traditional chemical pesticides (Zhang et al., 2019), thereby reducing environmental pollution and impact on human health. In terms of chemistry, extracts and essential oils from tobacco contain a wide range of chemical constituents (Bhisey, 2012; Zou et al., 2021) that can be widely used in industries such as food, flavouring and cosmetics to add unique flavours and functionality to products. In addition, compounds such as lignin and cellulose in tobacco can be converted into renewable energy sources (Grisan et al., 2016; Barla and Kumar, 2019; Srbinoska et al., 2021), such as bioethanol and biodiesel for power generation and transport through biomass energy technology, providing a new direction for the energy industry. These applications have enriched the utilisation value of tobacco, providing a diverse source of resources for medicine, agriculture, food and energy.

## 4 Characterization of morphological and compositional information

**FIGURE 2:**
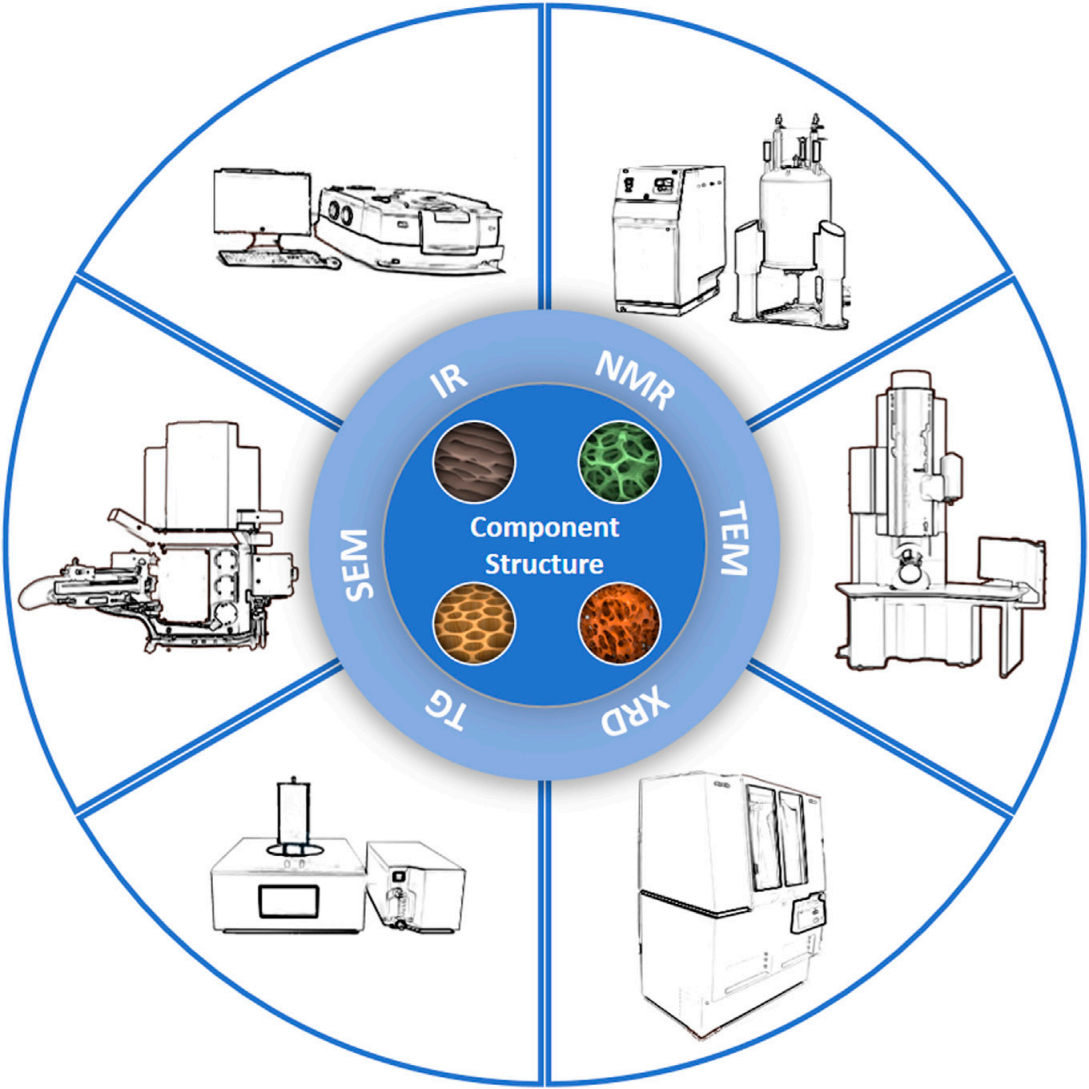
Ilustration showing the characterization techniques.

The composition of biomass profoundly influences the properties and applications of itself and its derivatives, such as combustion performance, catalytic performance and degradability. Therefore, advanced characterization techniques are needed to investigate the composition of biomass (Demirbas, 2004). Firstly, XRD can be utilized to study tobacco’s crystallinity. Secondly, biomass comprises cellulose, hemicellulose, and lignin, where NMR and high-performance liquid chromatography (HPLC) can elucidate tobacco’s molecular structure, providing references for further research into its derivatives. Morphology profoundly affects biomass storage, distribution, accumulation, and energy conversion efficiency within organisms (Leth-Espensen et al., 2020). On one hand, nitrogen adsorption-desorption and mercury intrusion porosimetry can be applied to analyze tobacco’s pore structure, furthering research into its applications in adsorption, catalysis, and energy fields. On the other hand, significant advancements in electron microscopy characterization techniques enable more intuitive observation of tobacco morphology through SEM and observation of its microstructure through TEM (Inkson, 2016; Yu et al., 2021), facilitating deeper research into related mechanisms. Finally, utilizing EDS technology combined with electron microscopy provides compositional information from specific local regions. Compared to other sample analysis methods, its advantage lies in the ability to select a particular area and visualize the distribution of components. We have summarized the characterization techniques and instruments related to tobacco discussed in this paper with a diagram, as shown in Figure 2. In summary, based on the above characterizations, opportunities are provided for a profound understanding of biomass’s basic structure and underlying mechanisms of related evolutions, thereby offering scientific references for more rational biomass applications.

### 4.1 Crystal phase—X-ray diffraction (XRD)

Natural polymeric compounds in biomass have unique structural characteristics such as crystal phases and crystallinity. XRD, which is the result of mutual interference between X-rays and crystalline samples, can be applied to analyze the natural polymeric compounds in biomass based on the positions and intensities of the diffraction peaks in the pattern, and thus infer the phase composition and crystallinity of the samples.

**FIGURE 3:**
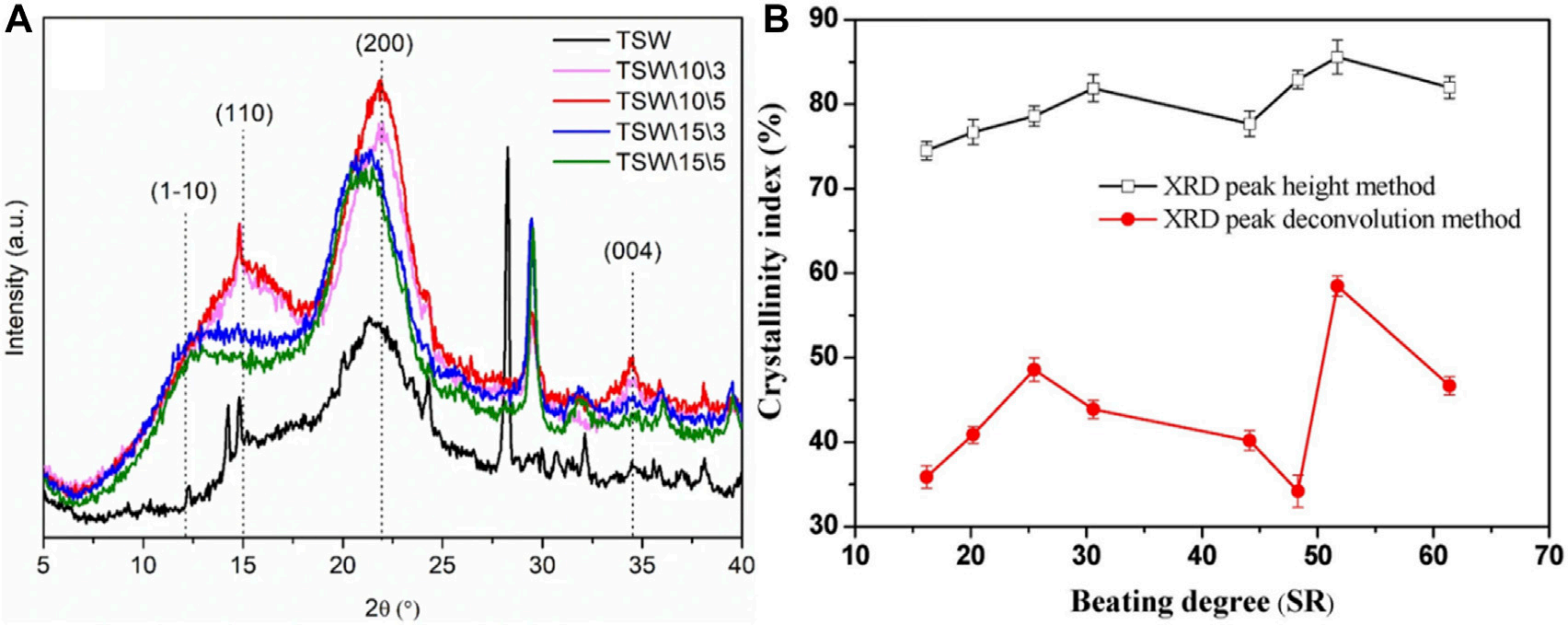
**(A)** X-ray differentiation of tobacco stalk wastes samples with and without alkaline treatment (reproduced with permission from Dallé et al. (2021)). **(B)** The Evolution of CrI of cellulose obtained from beaten tobacco stem pulp XRD patterns by peak height method (top) and deconvolution method (bottom) (reproduced with permission from Zhao et al. (2017)).

Each crystalline phase in an XRD pattern has a unique set of diffraction peaks and intensities, and diffraction peaks of different phases may overlap but do not interfere with each other. Dallé et al. investigated the changes in cellulose crystal type and crystallinity of tobacco straw waste (TSW) before and after NaOH treatment (exposure times of 3 and 5 h, respectively), as shown in Figure 3A (Dallé et al., 2021). Miller index of (110) is related to cellulose type I structure while miller index of (1–10) is related to cellulose type II. With 10% NaOH processing for 3 and 5 h (named TSW\10\3 and TSW\10\5, respectively), the cellulose type I structure is still maintained as the original TSW without NaOH treatment. After 15% NaOH treatment, miller index of (1–10) corresponding to cellulose type II is appeared. The results indicate that the conversion of cellulose I to cellulose II is higher at higher concentrations of NaOH solutions, a process that typically occurs during chemical treatment of natural fibers with NaOH due to hydrogen bonding reactions that lead to decrystallization and change the polycrystalline form of cellulose. This improves the properties of the cellulose and provides the basis for applications in tobacco stalks. Ning et al. studied the changes in the crystalline structure of samples of different tobacco forms after microbial fermentation and evaluated the effects of fermentation on these characteristics, laying the foundation for a better understanding of the correlation between microorganisms and tobacco quality (Ning et al., 2023).

XRD can be used for qualitative analysis of phases, determining grain size, lattice parameters, etc. In situations where the phase category is known, the phase can be quantitatively analyzed by measuring the integral diffraction intensity of phase diffraction peaks to estimate their relative contents. Zhao et al. studied the quantitative characterization of the crystal structure characteristics of plant cellulose fibers during the process, as shown in Figure 3B. The results showed that the change in the crystallinity index (CrI) during the beating process of cellulose presented a two-stage feature, namely, an initial increase followed by a decrease (Zhao et al., 2017). In the first beating stage, the increase in CrI may be due to the action of beating on the amorphous region of cellulose, leading to a reduction in the amorphous region and a relatively increase in the crystalline region. In the second beating stage, the slight increase in CrI may be related to the removal of lignin and hemicellulose from cellulose, and the subsequent downward trend may be due to the continued destruction of the crystal structure, providing a research basis for the effect of mechanical stirring on cellulose crystal structure (Zhao et al., 2017). Crystallinity calculated from XRD data needs to be background-calibrated to obtain an estimate, and the most widely used peak height method overestimates the true crystallinity (Barnette et al., 2011).

### 4.2 Molecular structure—nuclear magnetic resonance (NMR) and high-performance liquid chromatography (HPLC)

**FIGURE 4:**
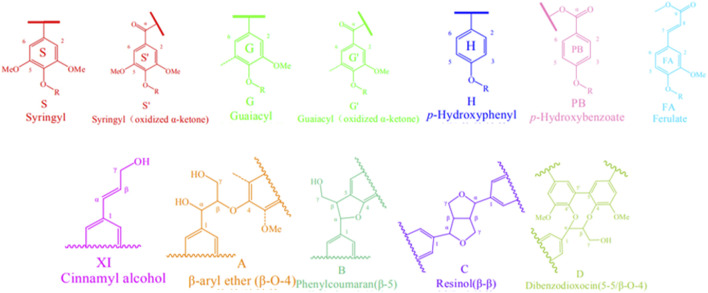
2D HSQC NMR spectra of lignin from middle leafs of flue-cured tobacco provide the molecular structures of lignin components (reproduced with permission from Miao et al.).

Biomass is composed with many moleculars, such as lignin, sugar and polyphenols. Among them, lignin is present in most terrestrial plants and is derived from hydroxycinnamyl alcohols and related compounds (monolignols). It is an oxidatively coupled aromatic biopolymer (Lee et al., 2019; Tobimatsu and Schuetz, 2019). Studies have shown that lignin accounts for 25%–30% of the biomass of most plants, making it one of the most abundant renewable resources on earth (Norgren and Edlund, 2014; Ragauskas et al., 2014; Schutyser et al., 2018). Lignin in tobacco is mainly found in tobacco leaves, where it is abundant and may serve as a valuable industrial material. It is not only used as a raw material for the production of second-generation lignocellulosic ethanol and biomass fuels but also possesses attractive properties such as antioxidant, UV-blocking, and antimicrobial activities (Shin et al., 2009; Kai et al., 2016; Roopan, 2017; Collett et al., 2019). Lignin in tobacco also has a significant impact on the quality and safety of tobacco products. In order to improve the quality of tobacco products, the team led by Gao explored the chemical structure and content of lignin in tobacco. Through two-dimensional heteronuclear single quantum coherence nuclear magnetic resonance (2D HSQC NMR) technology for structural characterization, they identified 10 major lignin basic units in tobacco lignin samples (Figure 4) and the bonding structures between units, along with signals for 27 related structures. In 2D HSQC NMR, the peaks of carbon and hydrogen nuclei directly connected to functional groups are correlated, greatly improving the resolution of the spectrum by obtaining hydrogen nucleus data, with sensitivity more than 30 times higher than traditional 13C NMR (Miao et al., 2022). Through semi-quantitative analysis of lignin in tobacco samples, it was found that the lignin in tobacco samples is of the SGH type, with guaiacyl units (G) being the predominant basic units, ranging from 50.72% to 73.50% in content, while syringyl units (S) range from 15.62% to 26.51%, and p-hydroxyphenyl units (H) range from 5.66% to 28.90%. The β-aryl ether unit β-O-4 (A) is the predominant interunit linkage, ranging from 72.93% to 92.97% in content, followed by the resinol unit β-β (C), ranging from 3.88% to 15.81% (Miao et al., 2022). 2D HSQC NMR technology provides significant assistance in the analysis of complex lignin structures in tobacco, as well as in the analysis of cellulose and hemicellulose, and even in elucidating the fundamental properties of tobacco biomass. It lays the groundwork for the analysis and application of lignocellulose in chemical engineering and energy fields.

**FIGURE 5:**
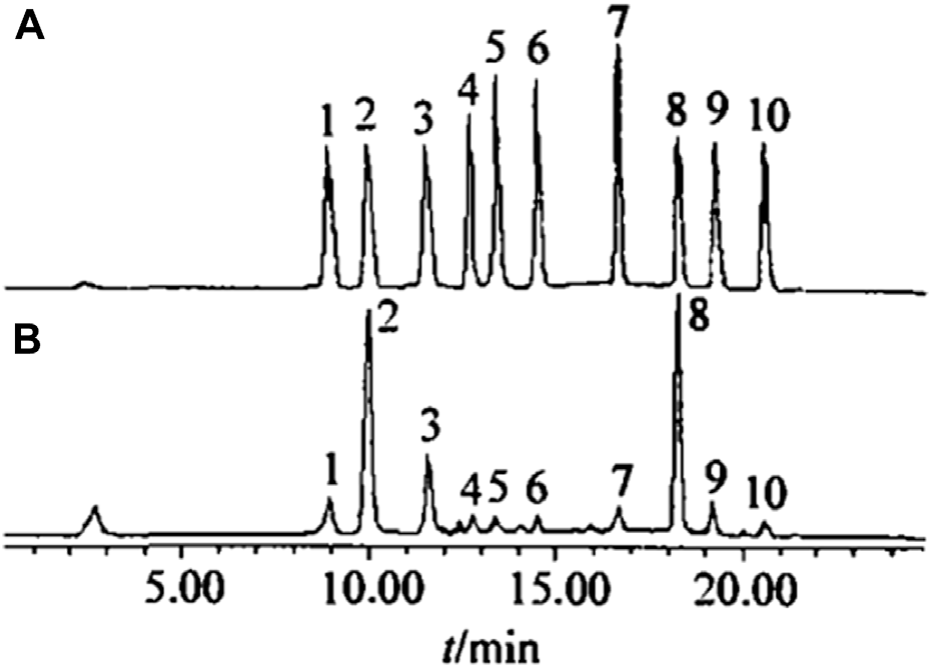
Chromatogram of standard sample **(A)** and tobacco sample **(B)** (1. 5-O-cafoylquinicacid; 2. chlorogenic acid; 3. 4-O-caffoylquinic acid; 4. caffeic acid; 5. esculetin; 6. scopoletin; 7.scopoltin; 8. rutin; 9. kaempferol-3-rutinoside; 10.quercitrin).

Polyphenolic substances in tobacco leaves mainly include chlorogenic acid, rutin, scopolamine, among others. The content of polyphenols varies with the genetic type of tobacco and cultivation conditions. It is generally believed that polyphenols make a significant contribution to the quality of smoke and are one of the key components in producing the aroma of tobacco smoke. Therefore, researching polyphenolic substances in tobacco can better complement and coordinate the chemical components of tobacco leaves of different regions, types, and grades, thereby obtaining products that meet quality requirements. Zhang Tian’s team used solid-phase extraction for preseparation and HPLC to determine ten kinds of plant polyphenols in tobacco samples, including 5-O-caffeoylquinic acid, chlorogenic acid, 4-O-caffeoylquinic acid, caffeic acid, esculin, scopoletin, scopolin, rutin, kaempferol-3-rutinoside, and quercitrin (Figure 5). HPLC, based on classical liquid chromatography, uses liquid as the mobile phase and employs a high-pressure liquid delivery system (Xiao and Oefner, 2001; Horváth, 2013). Different solvents with single polarity or different proportions of mixed solvents, buffer solutions, etc., are pumped into chromatographic columns containing extremely fine particles of efficient stationary phases (Xiao and Oefner, 2001; Horváth, 2013). Following the separation of components in the chromatography column, they proceed to the detector for subsequent analysis, thereby enabling comprehensive sample examination (Xiao and Oefner, 2001; Horváth, 2013). The standard recovery rate was 94%–105%; RSD was 1.3%–1.5%. This method was used to determine the ten kinds of plant polyphenols in tobacco samples, and the results were satisfactory.

Sugars are another important class of compounds in tobacco. Water-soluble sugars, especially reducing sugars, are closely related to the aroma and taste of tobacco. Sugar compounds are also the main precursors of harmful components such as tar, polycyclic aromatic hydrocarbons, and acetaldehyde in tobacco smoke (Sanders et al., 2003).

The HPLC-NMR coupling technique has matured with technological advances, but further optimisation is still required, especially to improve the sensitivity of NMR, e.g., for cellulose analyses, which can be interfered with by other non-cellulosic elements (Barnette et al., 2011).

### 4.3 Pore structure—gas adsorption and mercury intrusion porosimetry

Biomass has a natural porous structure. Due to its wide availability and low cost, biomass is considered an ideal precursor for the preparation of biomass-derived porous materials, such as porous carbon materials. Different biomasses could produce channels with different sizes for transporting water and nutrients, and the resulting porous carbon materials might retain these original different channel structures. Therefore, they could have various industrial applications due to these pores, such as adsorption, separation, catalysis, and so on. For more appropriate applications, the study of the pore structure of porous materials is very important.

According to the International Union of Pure and Applied Chemistry (IUPAC) standard (Sing et al., 1985), porous materials are classified into three categories based on pore size: micropores (<2 nm), mesopores (2–50 nm), and macropores (>50 nm). For the analysis of microporous or mesoporous pore structures, the gas adsorption method is commonly used (Barrande et al., 2009; Vogt et al., 2019; Benavente et al., 2021). Gas adsorbs on the surface of a solid adsorbent, preferentially entering smaller pores to form a monolayer adsorption. As the pressure increases, multilayer adsorption occurs. By measuring the volume of gas adsorbed at different pressures, an adsorption-desorption isotherm is obtained. This data, combined with theory, allows for the analysis of the porous material’s structure, providing information on the material’s specific surface area, pore volume, and pore size (Sing, 2001), with the Brunauer-Emmett-Teller (BET) theory being the most widely used.

Tobacco, as an important porous biomass material, has a pore structure that significantly affects its chemical and physical properties. In cigarette production, the moisture retention and equilibrium moisture content of tobacco are closely related to its pore structure (Lou et al., 2014; Yin et al., 2015). During the flavoring and casing process, the tobacco’s pore structure influences its adsorption properties for flavorings and fragrances (XueYi et al., 2012). Additionally, the pore structure of tobacco is an important factor affecting its combustion and pyrolysis performance (Guo et al., 2019). The adsorption gas commonly used for characterizing tobacco pore structure is nitrogen at a temperature of 77 K. For example, Liu et al. used the nitrogen adsorption method to measure the specific surface area and porosity of three different types of tobacco leaves (flavored tobacco, flue-cured tobacco, and burley tobacco), finding a clear visual difference in structure among them at the same sample volume: burley tobacco was more porous, flavored tobacco relatively dense, and flue-cured tobacco in between (Liu et al., 2015). In Section 2, Sha et al. obtained the specific surface area of porous carbon (1,104 m^2^ g^−1^) using the same method (Sha et al., 2015). Moreover, Tan et al. achieved excellent specific surface area performance of 2,749 m^2^ g^−1^ with porous carbon prepared from tobacco stems and leaves, laying the foundation for further applications in catalysis and energy (Tan et al., 2023). Furthermore, combining nitrogen adsorption-desorption with other analyses can correlate the material’s structure and properties. Yin et al. based on the BET theory, used nitrogen adsorption to measure the specific surface area and volume of different environmental flue-cured tobacco samples (Yin et al., 2015). Combined with chemical composition analysis, they found a direct connection between the physical structure and chemical composition of tobacco and moisture diffusion, indicating that the content of pectin, total sugars, water-soluble sugars, and specific pore volume significantly contribute to the effective diffusion coefficient of water.

**FIGURE 6:**
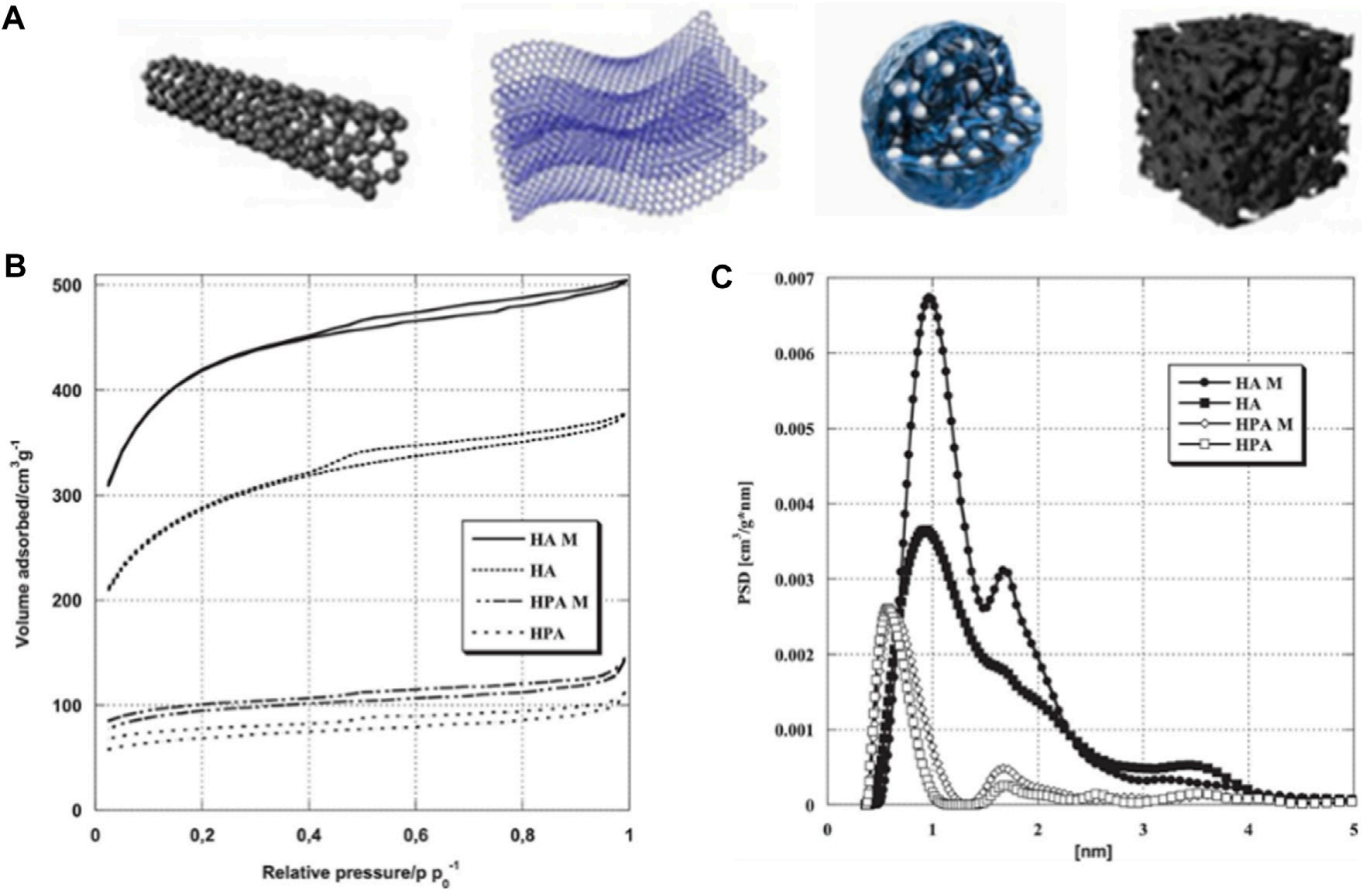
**(A)** Modes of carbon materials derived from biomass. **(B)** Nitrogen adsorption isotherms and **(C)** pore size distributions of porous carbons (reproduced with permission from Kazmierczak-Razna et al. (2017)).

The biomass-derived carbon materials are porous as well, there are various types of pore shapes, as shown in Figure 6A. Kazmierczak-Razna et al. used low-quality hay as raw material and activated the resulting char with phosphoric acid to produce phosphorus-containing carbon materials (Kazmierczak-Razna et al., 2017). They studied the effects of mild and conventional heating methods on the physicochemical properties of the materials. The BET method was used to characterize the materials’ porous structures with nitrogen adsorption, as shown in Figure 6B. All samples obtained Type I isotherms with small hysteresis loops, indicating the presence of pores with larger diameter, including mesopores of 2–4 nm, as well elucidated by the pore volume distribution function calculated by DFT (Figure 6C), which showed the differences in porous structure due to heating methods.

In the characterization of pore structure, the mercury intrusion method is considered as a complementary method to gas adsorption. The gas adsorption method measures relatively smaller pore sizes, while the mercury intrusion method covers a broader range of pore sizes (4 nm–400 μm). This method involves applying external pressure to force non-wetting liquid mercury into the sample’s pores, obtaining a curve of pressure versus mercury volume, thereby determining the pore size parameters of the sample (Dim et al., 2016). Xu et al. used the mercury intrusion method to investigate the changes in pore size distribution of flue-cured tobacco and burley tobacco during the drying process under different drying devices, such as drum and moving bed dryers (Xu et al., 2015). The results showed that the internal pore volume of the tobacco leaves shrank noticeably under different drying methods, and their volume shrinkage rate was linearly related to the moisture content. Guo et al. utilized the mercury intrusion method to measure the pore sizes of different types of tobacco, exploring the relationship between the tobacco’s pore structure and its physical moisture retention properties (Guo et al., 2019). They found that under the same conditions, the water loss rate was highest for reconstituted tobacco leaves and expanded stem, followed by burley and flavored tobacco, with flue-cured tobacco having the lowest water loss rate. Furthermore, the wettability of various tobacco samples was negatively correlated with the average pore size of their micropores. Gas adsorption and mercury compression can clearly measure the pore size distribution of materials, but there are some limitations, such as gas adsorption has a low measurement accuracy for large pore size materials, while mercury compression requires the use of toxic mercury as a medium, which makes it more dangerous to operate the process, and the fragile structure of the biomass is susceptible to damage when mercury enters its pores.

### 4.4 Morphology—scanning electron microscopy (SEM)

**FIGURE 7:**
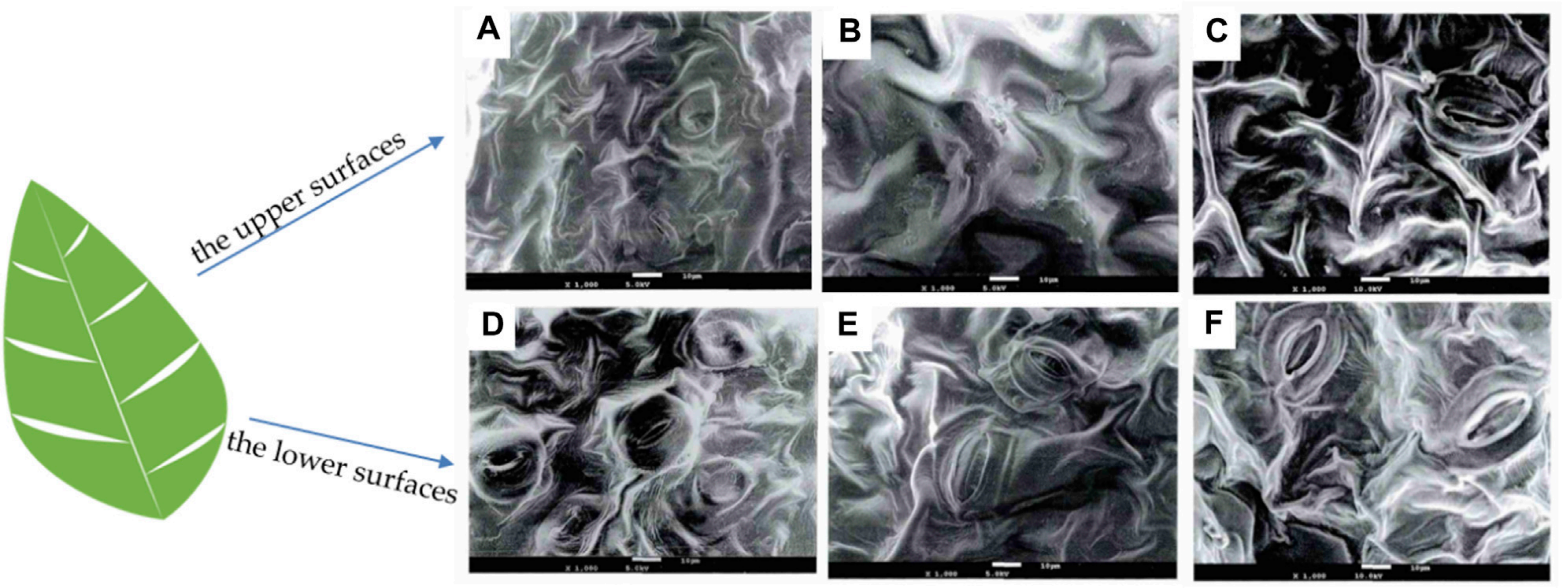
SEM images of **(A–C)** the upper surfaces and **(D–F)** the lower surfaces of spice, roasted, and white-ribbed tobacco samples, respectively (reproduced with permission from Yang et al. (2015)).

The morphology is an important parameter affecting the catalysis and optical performance of biomass and its derivatives. The morphology of biomass is greatly affected by the environment and could be inherited by the derivatives. It is necessary to investigate its morphological structure using advanced characterization tools. SEM is a technique that utilizes signals of secondary electrons and backscattered electrons to directly reflect the surface morphology and pore structure of biomass and biochar (Li et al., 2021). Yang et al. examined the upper and lower surfaces of various tobacco samples using SEM (Figure 7) discovering that the stomata of all three types of tobacco had similar shapes, with narrow fusiform stomatal slits surrounded by a heart-shaped ring of bulges (Yang et al., 2015). The study found that the upper surfaces of the three types of tobacco samples were smoother with larger pleats and gentler slopes, and had fewer stomata (Figures 7A–C). In contrast, the lower surfaces had smaller and denser pleats, more stomata (Figures 7D, E) (Yang et al., 2015). It is hypothesized that the variation between the upper and lower surfaces of the leaves may be linked to photosynthesis. The upper leaves need to participate in more photosynthesis while the lower leaves less.

Biochar obtained from different heating temperatures exhibits varying morphologic structures. Sha et al. (Sha Y. et al., 2015) observed the structure of N-doped porous carbons prepared from waste tobacco using SEM, which reveals that porous carbons obtained from 500°C exhibited a smooth surface, while 700°C and 900°C showed increased surface roughness. SEM allows observation of biomass, but only the surface morphology of the sample can be observed, while the structure below the surface cannot be detected.

### 4.5 Microstructure—transmission electron microscopy (TEM)

**FIGURE 8:**
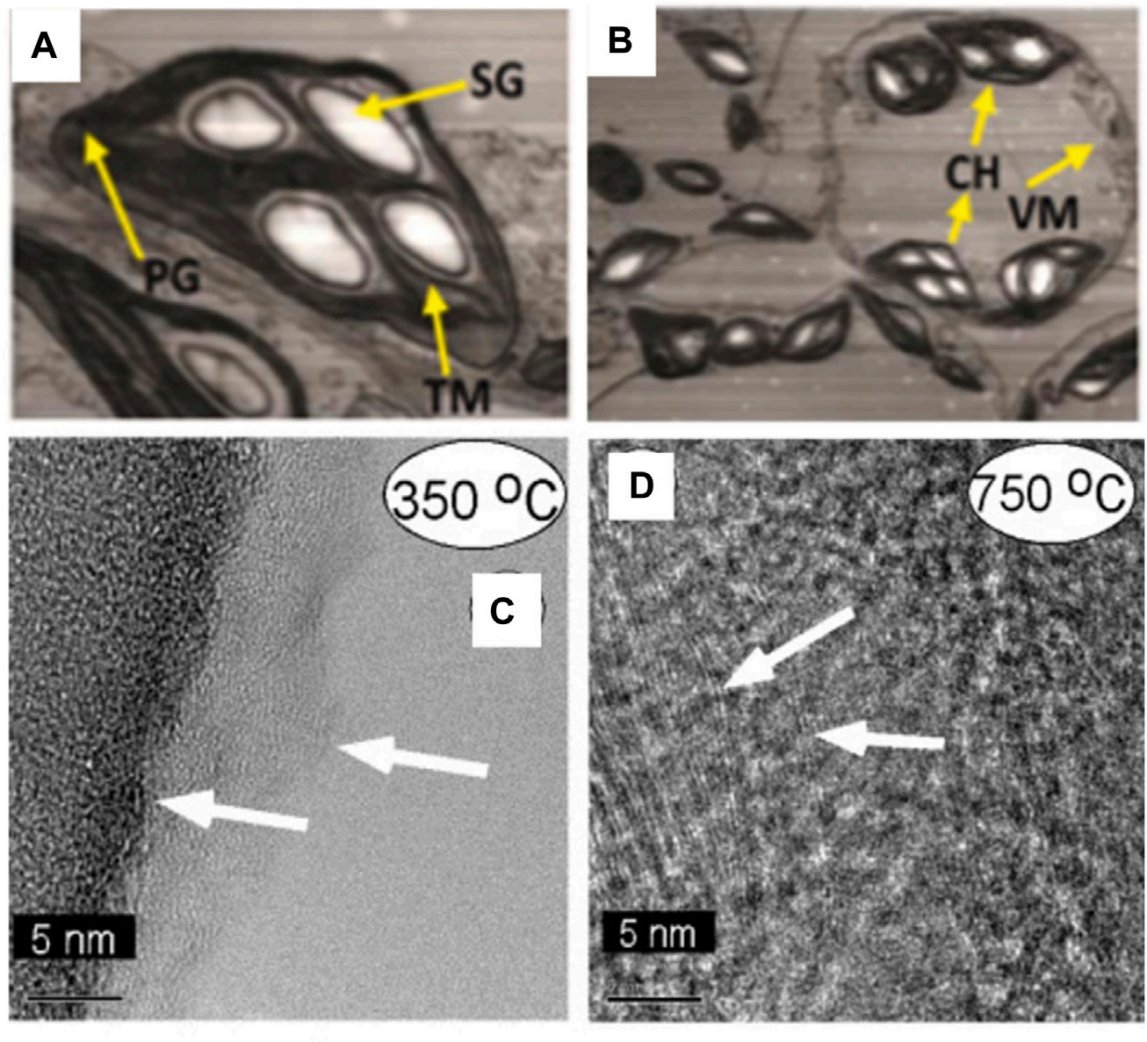
**(A, B)** TEM observation of N.benthamiana leaf tissues infected with TMV (SG, starch granule; PG, plastoglobule; TM, thylakoid membrane; VM, vacuole membrane; CH, chloroplast) (reproduced with permission from Adeel et al. (2021)). **(C)** TEM images showing much of this sample displayed a homogeneous, amorphous microstructure (arrows) at 350°C. **(D)** TEM images showing graphite sheets is formed at 750°C (marked in left side of image with arrows) and the sample was heterogeneous, with mixed microstructures ranging from amorphous to graphitic (reproduced with permission from Baliga et al. (2003)).

The microstructure of biomass plays a crucial role in determining its properties and functionalities, such as strength and adsorption capabilities. Consequently, researchers have increasingly focused on investigating the microstructure of biomass. TEM enables the examination of ultrastructural changes in biological samples under varying conditions, shedding light on modifications in organelle structures and facilitating the study of cell organelle alterations. Adeel et al. observed significant ultrastructural changes in N.benthamiana leaf tissues following tobacco mosaic virus (TMV) infection using TEM (Adeel et al., 2021). The study revealed the emergence of multiple large starch granules, anomalous distribution of plastoglobules, distortion of thylakoid membranes, disruption of grana stalks, and invagination of vesicular membranes (Figure 8A). They also noted a reduction in the number of chloroplasts compared to healthy controls, exhibiting irregular sizes and shapes, providing visual evidence for exploring leaf cell responses to viral infections (Figure 8B).

The transformation of the carbon matrix derived from tobacco at different temperatures showcases diverse microscopic details, with TEM offering a valuable tool for visualizing the microstructure of smoky charcoal. Baliga et al. examined the morphological changes of pyrolyzed tobacco and its constituents—cellulose, pectin, and lignin—in a helium environment (Baliga et al., 2003). High-resolution TEM characterized the carbon matrix generated from tobacco and pectin charcoal. As depicted in Figure 8C, the sample displayed a uniformly amorphous nature after undergoing high-temperature treatment at 350°C. With increasing temperature, the presence of graphitic flakes gradually intensified. At 750°C, these graphitic flakes were embedded within nearly amorphous material, transitioning into a non-homogeneous mixed microcrystalline structure from amorphous to graphiti, illustrated by the arrows in Figure 8D. Furthermore, Miao et al. directly observed the morphology and particle size distribution of C-dots prepared from tobacco using TEM. They found that the C-dots existed in sizes of 2.14 ± 0.3 nm without aggregation, demonstrating satisfactory uniformity (Miao et al., 2018).

**FIGURE 9:**
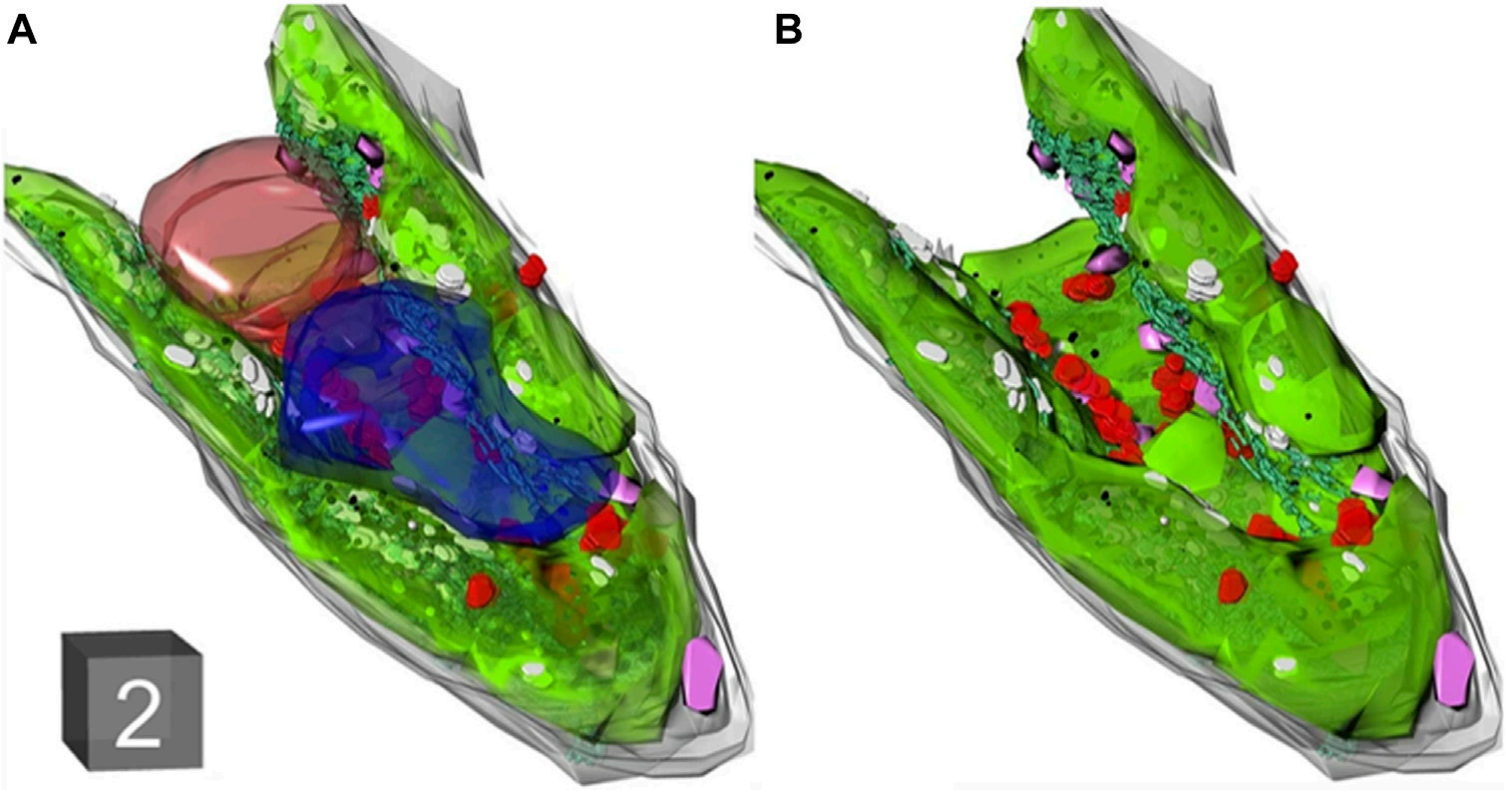
**(A, B)** 3D reconstruction of tobacco leaf cell imaged with TEM. Cell wall (gray), chloroplasts (green), mitochondria (red), nucleus (brown), peroxi-somes (purple), and vacuoles (blue). Cube = 2 μm^3^ in **(A)** (reproduced with permission from Zechmann et al. (2022)).

Three-dimensional imaging of tobacco cells using SEM and TEM can yield volumetric data on organelles, enabling a comprehensive visualization of tobacco ultrastructure. Zechmann et al. employed TEM to reconstruct tobacco cells from 71 sections, revealing that vesicles dominate approximately 70% of the total cell volume (Figure 9) (Zechmann et al., 2022). Despite the advancements, conventional microscopy still leaves certain aspects of plant meiosis unexplored. Addressing this gap, Mursalimov et al. utilized serial block-face scanning electron microscopy (SBF-SEM) to delve into tobacco male meiosis, facilitating three-dimensional ultrastructural analysis (Mursalimov et al., 2021). Their discoveries highlighted nucleus migration in 90%–100% of tobacco meiotic cells, significantly advancing plant meiosis research. Notably, all assessments presented are objective and backed by evidence. TEM has a high resolution for revealing the microstructure, however the development of low electron dose transmission electron microscopy techniques is necessary because the samples required for TEM are thin and highly susceptible to damage to the sample structure, especially biomass is generally sensitive to electron beams.

### 4.6 Elements analysis—energy dispersive X-ray spectroscopy (EDS)

Biomass is rich in elemental species, such as carbon, oxygen and hydrogen. Processing and the induction under different conditions produce corresponding changes in species and contents, which in turn affects the form and content of elemental species in biomass and its derivatives, and further affect the performance. Therefore, it is of great importance to study the elemental species and their contents. EDS, as one of the elemental analysis methods, can be used to examine the elemental composition and content of samples to obtain more comprehensive compositional information (Abd Mutalib et al., 2017). To extract information on specific surfaces and areas, EDS is generally used in conjunction with SEM and TEM, which have local feature. Precisely because of this, EDS has advantages that other compositional analysis methods lack; it can analyze both the structure and the composition simultaneously, even achieving nanometer-level resolution. Additionally, it can investigate the local composition to explore its distribution uniformity.

**FIGURE 10:**
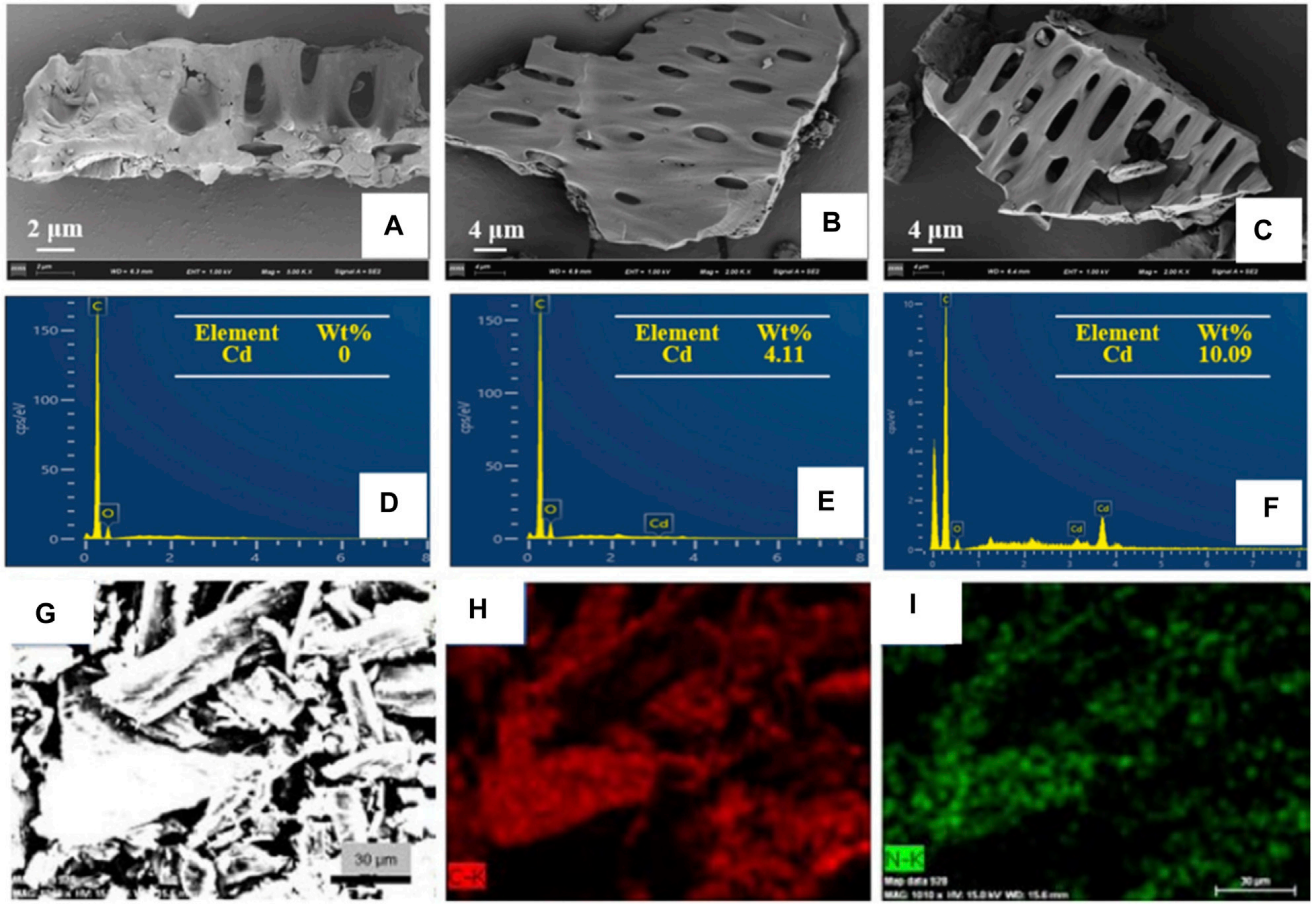
**(A–C)** The SEM images and **(D–F)** EDS spectra of biochar that produced from waste tobacco before and after Cd^2+^ ion adsorption (reproduced with permission from Yu et al. (2021)). **(G–I)** SEM image and corresponding elemental mapping of C and N elements of porous carbon that produced in 700°C calcination of waster tobacco (reproduced with permission from Sha et al. (2015)).

Yu et al. investigated the surface morphology changes of biochar after Cd^2+^ ion adsorption, as well as the corresponding changes in adsorption capacity (Yu et al., 2021). They found that the surface of biochar became rough due to corrosion by heavy metal ion adsorption. A new characteristic peak of Cd was observed in the EDS spectrum (Figures 10A–F), which was absent in that of raw biochar, indicating Cd^2+^ ion adsorbed on the tobacco stalk was converted onto biochar. This phenomenon provides evidence for studying the mechanism of Cd^2+^ adsorption by biochar. Sha et al. observed the elemental distribution of nitrogen-doped porous carbon, which were prepared from waste tobacco by a simple melamine pretreatment process and calcination (Sha Y. et al., 2015). The 19.08 wt% of N content and well-distribution in waste tobacco suggests that pre-treatment of the melamine process is a feasible way to produce fortified material from natural crops (Figures 10G–I), which makes a transition from useless to useful.

Biomass combustion produces a large amount of gaseous and particulate pollutants. Especially when incomplete combustion occurs, a large amount of particulate matter (PM), CO and polycyclic aromatic hydrocarbon are released (Bignal et al., 2008; Yao et al., 2023). The main component of particulate pollutants is carbonaceous particles, which greatly affect the health of human beings and thus it is of great importance to study the elemental composition and size. Slezakova et al. analyzed the chemical composition and morphological parameters of 4,000 individual particles by SEM combined with X-ray microanalysis, which provided specific information on the elemental composition of individual particles as well as their size and morphology (Slezakova et al., 2011). The effect of tobacco smoke on PM was investigated and the results showed that tobacco smoke mainly leads to PM2.5. EDS is able to measure heavy elements with high accuracy, but not for light elements, so it has some limitation for biomass consisting of a large number of light elements such as C, H, and O.

## 5 Characterization for pyrolysis process of tobacco

Studying dynamic processes is crucial for rational biomass utilization. Combining thermogravimetry and infrared spectroscopy, tobacco’s pyrolysis processes are characterized, which deepens the understanding of their dynamic nature and improving tobacco biomass resource development and energy utilization efficiency (Marcilla and Berenguer, 2023).

**FIGURE 11:**
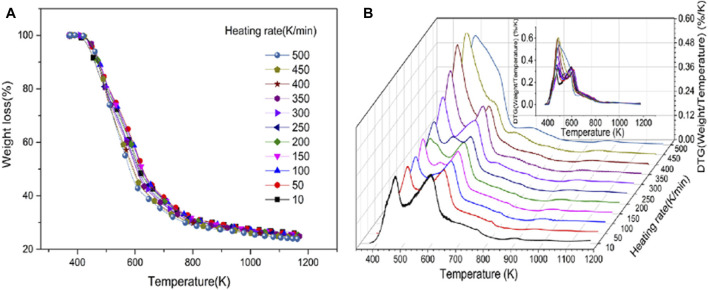
The TGA results of tobacco samples at 11 heating rates. The heating rates range from 10 to 500 K/min, with intervals of 50 K/min. Different heating rates **(A)** TGA curves and **(B)** DTG curves (reproduced with permission from Mu et al. (2022)).

Pyrolysis is one of the most widely used thermal conversion techniques for biomass transformation, capable of decomposing biomass into solid biochar, liquid bio-oil, and combustible gas to meet different needs. Thermogravimetric analysis (TGA) is a thermal analysis method that measures the relationship between the mass of a substance and temperature or time under a controlled temperature program in a certain atmosphere (Loganathan et al., 2017). It uses thermogravimetric curves (TG curves) and differential thermogravimetric curves (DTG curves) obtained during the process to interpret the physical or chemical changes in the thermal reaction process of the substance (Guo G. et al., 2019). This method, known for its simple principle and sensitive detection, has been widely applied in the qualitative and quantitative analysis of biomass pyrolysis processes, as well as the determination of kinetic parameters. It is also involved in the tobacco industry. For example, Mu et al. used TGA to study the pyrolysis characteristics of tobacco at a wide heating rate (10–500 K/min). The TG curve in Figure 11A shows that tobacco begins to pyrolyze at 373.15 K and completes pyrolysis at 1,173.15 K. Analysis of the DTG curve in Figure 11B reveals that the peak of the curve aggregates as the heating rate increases. Then, by deriving and calculating the TG/DTG curves for each component, kinetic parameters such as the mass fraction of each pyrolysis product, pre-exponential factor, and activation energy were obtained, finding that the activation energy of each reaction component is not sensitive to the heating rate (Mu et al., 2022). Traditional TGA has certain limitations in measuring large particles due to the influence of heat and mass transfer performance on pyrolysis analysis. Therefore, Guo et al. proposed a method based on macro thermogravimetric analysis (MTGA) to explore the pyrolysis differences among different tobaccos (Guo et al., 2022). This method characterizes differences using the root mean square error of the differential thermogravimetric curves, comparing MTGA and TGA for tobaccos from different parts, provinces, grades, and years. It was found that MTGA has the characteristics of fast identification speed and strong identification capability, able to accurately characterize the pyrolysis differences of different tobacco samples.

In practical material analysis, TGA is often used in conjunction with other analytical techniques to provide a more comprehensive and accurate characterization of material properties. Peng et al. utilized thermogravimetric-mass spectrometry (TG-MS) and pyrolysis-gas chromatography/mass spectrometry (Py-GC/MS) techniques to analyze the effect of oxygen on the pyrolysis process of tobacco (Peng et al., 2021). They found that the introduction of oxygen changed the chemical structure of tobacco, making it more prone to deoxygenation reactions and resulting in the formation of more ordered char surfaces. Besides being used with mass spectrometry, TGA can also be coupled with Fourier-transform infrared spectroscopy. Barontini et al. used thermogravimetric-Fourier transform infrared (TG-FTIR) spectroscopy to qualitatively and quantitatively analyze the volatile substances produced during the pyrolysis of tobacco (Barontini et al., 2013). They obtained quantitative data for key volatile substances such as CH_3_CHO, CO_2_, CO, nicotine, and water. They characterized the glycerol produced during pyrolysis based on a fixed-bed reactor, thus obtaining a complete compositional diagram of the gases formed during the pyrolysis of tobacco in nitrogen and air.

Infrared absorption spectroscopy is a commonly used method for determining and quantitatively analyzing substances. Its principle lies in the interaction between infrared light and the molecules of the sample being tested. This interaction causes changes in molecular dipole moments due to vibration or rotation, leading to transitions of vibrational and rotational energy levels from the ground state to the excited state, thereby forming molecular absorption spectra (Movasaghi et al., 2008). Qualitative analysis is carried out by unfolding the position and shape of the spectrum based on the characteristic absorption frequencies of functional groups, used for identifying known substances and detecting functional groups contained in unknown substances. Quantitative analysis of the sample is performed based on the intensity of characteristic peaks, thereby determining the molecular structure of the substance (Movasaghi et al., 2008).

Tobacco belongs to the Solanaceae family, and there are significant differences in the chemical composition of tobacco from different regions and varieties. Wang et al. studied the gases released during the pyrolysis of cigar filler tobacco (CFT), cigar wrapper tobacco (CWT), and flue-cured tobacco (FCT) using FTIR (Wang et al., 2021). They found that CFT released less CO_2_ compared to CWT, and the amount of CO_2_, CH_4_, CO, and aromatic compounds released by flue-cured tobacco was lower than that of cigar tobacco. Zhou et al. studied the pyrolysis of regenerated tobacco slices (RTS) modified with melamine phosphate (MP) prepared by the papermaking process (Zhou et al., 2013). The 3D FTIR spectra (absorbance-wavenumber-minutes) of the gas products obtained during the pyrolysis of pure RTS and MP-modified RTS showed that the main gases released were H_2_O, CO_2_, CO, NH_3_, carbonyl compounds (e.g., aldehydes, ketones, and acids), alcohols, phenols, alkanes, and alkenes. At temperatures above 400°C, the condensation of melamine phosphate groups in MP gradually increased with the elimination of NH_3_, leading to a sharp increase in NH_3_ release intensity in MP-modified RTS, far exceeding that of pure RTS, which would affect the release of fuel gases and the formation of coke during RTS pyrolysis (Zhou et al., 2013). This provides reference for studying the mechanism of modified tobacco. Wu et al. studied the effect of glycerol addition on the pyrolysis characteristics and product distribution of cigar tobacco (CT). FTIR analysis showed characteristic bands related to vibrational modes of functional groups such as -OH, -CH_2_/-CH_3_, C=C, and C-O, and NH_3_ was identified at a low absorption band. At the same time, they found that the absorption intensity of functional groups changed with temperature during pyrolysis, indicating that glycerol may lower the maximum release temperature and absorption intensity of pyrolysis products. Methane and aromatic hydrocarbons formed by lignin decomposition showed two-stage release characteristics for CT-G, which was different from CT, indicating a significant influence between glycerol and lignin. Glycerol not only affects the pyrolysis characteristics but also affects its gas release behavior (Yu et al., 2021; Wu et al., 2022). FTIR analysis can provide a deeper and more intuitive explanation of the characteristics and changes during the pyrolysis of tobacco leaves, providing a basis for related principles research.

**FIGURE 12:**
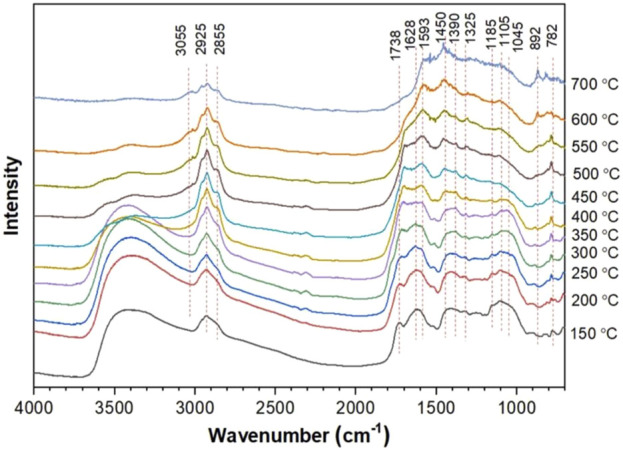
In-situ FTIR spectra of pyrolysis char during pyrolysis at different temperatures (reproduced with permission from Zhu et al. (2022)).

**FIGURE 13:**
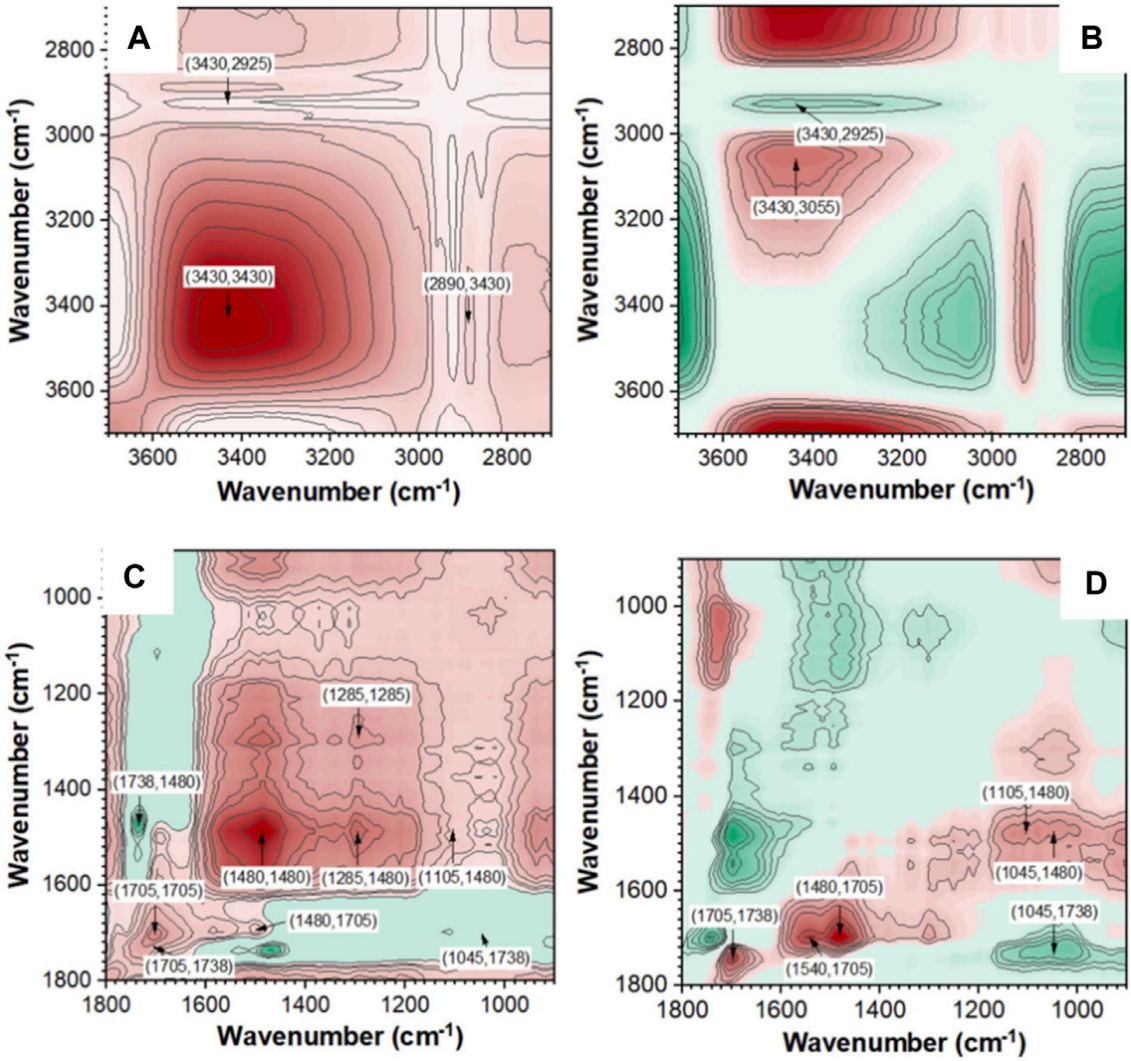
2D-PCIS synchronous and asynchronous spectra of pyrolysis char (reproduced with permission from Zhu et al. (2022)).

In addition, FTIR spectra can also be used to identify and analyze surface functional groups of samples. Yu et al. analyzed the surface functional groups of tobacco stalks transformed into biochar (TS-biochar) before and after adsorbing Cd^2+^ (Yu et al., 2021). They found that after adsorption, the peak at 1,546 cm^−1^ in the FTIR spectrum shifted to the right and increased in intensity, indicating the complexation of carboxyl groups with Cd^2+^. There were significant differences in the shape of the OH band before and after Cd^2+^ adsorption, revealing the interaction between OH and Cd^2+^. These results indicate that the complexation of oxygen-containing functional groups (OH, C=O, and COOH) with Cd^2+^ is an important way for TS-biochar to immobilize heavy metals, achieving long-term stable restoration of heavy metal-polluted soils. The development of *in-situ* infrared spectroscopy has made dynamic studies feasible (Yu et al., 2021). Zhu et al. studied the structural properties of pyrolysis residues and the reaction sequence of typical functional groups using *in-situ* Fourier transform infrared and two-dimensional correlation infrared spectroscopy (2D-PCIS) during pyrolysis (Zhu et al., 2022). Figure 12 shows the *in-situ* FTIR spectra of pyrolysis char during pyrolysis at different temperatures (Zhu et al., 2022). Figure 13 shows the synchronous and asynchronous spectra of pyrolysis char undergoing pyrolysis at the target temperature (Zhu et al., 2022). To study the evolution of molecular structure behavior in detail, FTIR bands in the ranges of 3,700–2,700 cm^-1^ and 1,800–900 cm^−1^ were analyzed. Negative crossover peaks indicate that the trend of carbonyl C=O in ketones differs from the C-H bending of CH_2_/CH_3_ and the C-O bending of alcohols. Positive and negative crossover peak results suggest that the dehydration and ring-opening reactions of polysaccharides occur earlier than the decomposition of carbonyl compounds such as ketones, esters, and acids (Zhu et al., 2022). Through the analysis of synchronous and asynchronous spectra and the evolution of surface functional groups of pyrolysis char, it is inferred that the hydrogen bond cleavage and dehydration of pyran rings occur simultaneously during low-temperature pyrolysis (Zhu et al., 2022).

The calibration of FTIR for quantitative analysis is primarily achieved through various mathematical methods, studying the relationship between the concentration of substances (or other physicochemical properties) and the response of the analytical instrument. The Lambert-Beer law can be used for qualitative and quantitative analysis of organic compounds, as well as for the analysis of unknown substances. In addition to the simplest Lambert-Beer law, there are two commonly used simple linear processing methods based on the least squares principle: one is called the Least Squares Method (CLS), and the other is called Multiple Linear Regression (MLR). Common nonlinear quantitative calibration models include Nonlinear Least Squares Model (NLS), Artificial Neural Network Model (ANN), and Support Vector Machine Model (SVM), among others (Mosorov, 2017). Barontini et al. obtained emission curves and quantitative data for key compounds in tobacco pyrolysis under different experimental conditions using advanced multivariate deconvolution techniques, but further exploration is needed for quantitative characterization of volatile product mixtures (Barontini et al., 2013). Zhao et al. studied the changes in cellulose crystal type and crystallinity during the mechanical pulping process of tobacco fibers using second derivative FTIR spectra and deconvoluted spectra in the OH stretching vibration region, revealing changes in hydrogen bond mode structure, including hydrogen bond energy, distance, and hydrogen bond content (Zhao et al., 2019). The results showed that, in addition to bond distance, both hydrogen bond energy and hydrogen bond content changed significantly with the increase in pulp beating, indicating that in addition to the effects of water, hydration, and swelling, the changes in cellulose hydrogen bond mode structure during mechanical pulping are closely related to internal/external fibrillation and stratification processes, providing a scientific basis for the deep mechanism of leaf refining (Zhao et al., 2019).

Combining all the above content, we have summarized the characterization techniques of tobacco and its derivatives, along with their corresponding contents, which are summarized in Table 2.

**TABLE 2:** Characterisation of tobacco derivatives.

Techniques	Materials and chemicals	Contents	References
Gas adsorption and mercury intrusion porosimetry	Porous carbon materials	Pore structure	Lou et al. (2014), Yin et al. (2015)
Scanning electron microscopy (SEM)	Tobacco samples and porous carbons	Surface morphology and pore structure	Li et al. (2021b)
Transmission electron microscopy (TEM)	Tobacco leaf tissues; cellulose; pectin; lignin	Microstructure	Adeel et al. (2021)
X-ray diffraction (XRD)	Cellulose	Crystal phase	Dallé et al. (2021)
Nuclear magnetic resonance (NMR)	Lignin; cellulose; hemicellulose	Molecular structure	Miao et al. (2022)
High-performance liquid chromatography (HPLC)	Polyphenolic substances	Molecular structure	Xiao and Oefner (2001), Horváth (2013)
Energy dispersive X-ray spectroscopy (EDS)	Porous carbon	Elements analysis	Sha et al. (2015)
Thermogravimetric analysis (TGA)	Tobacco	Characterization for pyrolysis process	Mu et al. (2022)
Fourier transform infrared spectroscopy (FTIR)	Biochar (TS-biochar)	Identify and analyze surface functional groups of samples	Yu et al. (2021)

## 6 Conclusion

In conclusion, the research contribution in developing characterization techniques for tobacco and its derivatives has been briefly summarized. Typical examples are illustrated to demonstrate the corresponding structure and composition features. In addition, the study progress in combustion and pyrolysis products of tobacco is also present. For making further achievement in characterizing the biomass and its derivatives, future research directions for this field are proposed as follows. Firstly, there will be a growing interest on developing *in situ* and operando characterization methods to monitor dynamic processes in real time from biomass to its derivatives. Furthermore, researchers may explore new spectroscopic and imaging techniques to probe the interactions between biomass components and nanomaterials, advancing our understanding of complex materials systems. Future research also waits for computational modeling and artificial intelligence for further predictive analytics and biomass-derived development.
